# Adverse events of givosiran in the treatment of acute hepatic porphyria: a pharmacovigilance study using the FAERS and VigiAccess databases

**DOI:** 10.1186/s13023-026-04446-8

**Published:** 2026-06-10

**Authors:** Yinpeng Xu, Fang Li, Li Huang, Mei Zhang, Pengqiang Du

**Affiliations:** 1 Pharmacy Department, Zhengzhou Ninth People’s Hospital, Henan, China; 2 Pharmacy Department, Women and Infants Hospital of Zhengzhou, Henan, China; 3 Pharmacy Department, Fuwai Central China Cardiovascular Hospital, Zhengzhou, Henan China

**Keywords:** Givosiran, Acute hepatic porphyria, Pharmacovigilance, FAERS, VigiAccess

## Abstract

**Background:**

Acute hepatic porphyria is a rare metabolic disorder characterized by life-threatening acute attacks and chronic neurological symptoms. Givosiran is an RNA interference therapeutic approved by the FDA in 2019 to treat AHP by inhibiting hepatic ALAS1 expression. This study aims to provide a comprehensive pharmacovigilance analysis of givosiran to evaluate its safety profile in real-world clinical settings.

**Methods:**

A retrospective disproportionality analysis was conducted using the FAERS database (from inception to Q4 2025) and the VigiAccess database (from inception to January 2026). Four algorithms—ROR, PRR, EBGM, and BCPNN—were employed to detect positive signals. Secondary analyses included clinical prioritization of signals and TTO analysis using the Weibull distribution test.

**Results:**

Overall,1,286 adverse event reports identifying givosiran as the primary suspect were extracted from FAERS, while the external validation using VigiAccess retrieved 1,326 reports. Among the FAERS reports with available data, female patients were more frequently reported, however, most cases lacked gender information. This demographic trend was supported by VigiAccess data, where reports were predominantly from the Americas (1,024 cases) and Europe (297 cases), with females accounting for 189 cases. Disproportionality analysis identified several adverse events consistent with previously recognized risks, including increased blood homocysteine, renal impairment, and pancreatitis. In addition, multiple signals not currently included in the product labeling—such as abdominal pain, seizure, and mental disorder—were consistently detected across both databases and warrant further investigation. Clinical prioritization categorized acute pancreatitis, seizure, abdominal pain, and renal impairment as moderate priority signals. TTO analysis revealed a mean onset of 544.36 days, with a Weibull shape parameter (β) of 1.01 (95% CI: 0.73–1.26), indicating a random failure-type risk pattern where the hazard remains constant over time. Sensitivity analysis excluding several PTs potentially related to the underlying manifestations of acute hepatic porphyria yielded consistent results, with major metabolic, pancreatic, and hepatic signals remaining statistically significant. Validation in VigiAccess identified 35 signals, confirming key risks such as blood homocysteine Increased and pancreatitis.

**Conclusions:**

This study corroborates the known risks of givosiran, including elevated blood homocysteine and renal impairment, using both the FAERS and VigiAccess databases, and identifies significant associations of givosiran with pancreatitis and seizures. The consistent detection of these signals across two independent databases further supports their credibility. The random failure-type risk pattern indicates that continuous safety monitoring should be maintained throughout the entire treatment course.

**Supplementary Information:**

The online version contains supplementary material available at 10.1186/s13023-026-04446-8.

## Introduction

Acute Hepatic Porphyria (AHP) is a rare metabolic disorders resulting from hereditary defects in the hepatic heme biosynthetic pathway [1–3]. The disease is characterized by acute and chronic symptoms arising from the involvement of both the central and peripheral nervous systems. While acute attacks typically persist for 3 to 5 days, symptoms in some patients may endure for 1 to 2 weeks or longer [4, 5]. Research indicates that AHP affects individuals across all ethnic and racial groups [6], with approximately 90% of symptomatic cases occurring in women [7].The clinical manifestation of acute attacks requires the induction of δ-aminolevulinic acid synthase 1 (ALAS1), which serves as the initial and rate-limiting enzyme in hepatic heme synthesis. Givosiran is an RNA interference therapeutic designed to treat AHP by inhibiting hepatic ALAS1 expression. Approved by the U.S. Food and Drug Administration (FDA) in 2019, givosiran significantly reduces the levels of ALAS1 and porphobilinogen (PBG), the primary pathological biomarkers of AHP [8].

Although givosiran has demonstrated significant efficacy in reducing the frequency of acute attacks in patients with AHP, current knowledge regarding its safety profile is largely derived from clinical trials and limited observational studies, including elevations in liver enzymes, renal dysfunction, hyperhomocysteinemia, and pancreatitis. However, their relatively small sample sizes, strict inclusion criteria, and limited follow-up periods may restrict the detection of rare, delayed, or unexpected adverse events. Therefore, post-marketing pharmacovigilance studies based on large real-world databases are essential to comprehensively characterize the safety profile of givosiran.

Spontaneous reporting systems, including the U.S. Food and Drug Administration Adverse Event Reporting System (FAERS) and the World Health Organization global pharmacovigilance database (VigiAccess), have become valuable resources for detecting potential drug safety signals in real-world clinical practice [9, 10]. Data-mining approaches applied to these databases have been widely used to identify previously unrecognized adverse drug reactions and to assess the safety of newly approved therapies [11, 12]. Nevertheless, comprehensive pharmacovigilance analyses specifically focusing on givosiran remain limited, and studies simultaneously validating safety signals across multiple international databases are particularly scarce. To address these gaps, the present study performed a comprehensive pharmacovigilance analysis of givosiran-associated adverse events using the FAERS database, with external validation in the VigiAccess database. This study aims to provide updated real-world evidence to support safer clinical use of givosiran in patients with acute hepatic porphyria.

## Materials and methods

This study was designed as a retrospective pharmacovigilance disproportionality analysis. The primary analysis utilized the FAERS database to calculate disproportionality signals (ROR, PRR, EBGM and BCPNN) for givosiran. Secondary analyses included descriptive statistics of the cases, time to onset (TTO) analysis, sensitivity analysis and a clinical prioritization of the detected signal. Furthermore, the discussion includes a review of literature regarding potential mechanisms.

### Source of data

In addition to the primary FAERS database, the VigiAccess database was used as a supplemental data source to support the reproducibility of the positive signals. This FAERS dataset spanned from the inception to Q4 2025, and the VigiAccess dataset spanned from the inception to January 2026.

### Data processing

Data extraction and preprocessing were performed using structured queries based on the generic and brand names of the drug (“givosiran” and “givlaari”). FAERS data were obtained in quarterly files and integrated into a unified dataset. Deduplication followed FDA-recommended methods: Reports were selected based on PRIMARYID, CASEID, and FDA_DT from the DEMO table, then sorted by CASEID, FDA_DT, and PRIMARYID. For reports with identical CASEIDs, retain the report with the largest FDA_DT value. For reports with identical CASEID and FDA_DT values, retain the report with the largest PRIMARYID value. Filter to retain data where the primary suspected (PS) drug is listed in the “Role_Code”field. Additional variables, including age, sex, and country, were used to verify potential duplicates and ensure data consistency. VigiAccess data were obtained from the WHO global pharmacovigilance database through its public interface (https://www.vigiaccess.org), which provides aggregated safety reports from VigiBase. As VigiAccess provides only aggregated data, individual case-level analyses could not be performed using this database. Data extraction was performed independently by two investigators to ensure accuracy and consistency. All adverse events were coded according to MedDRA version 27.1 at the Preferred Term (PT) level. Cases in which givosiran was identified as the primary suspect drug were included in the analysis.

### Data analysis

The overall analytical workflow included data extraction, deduplication, descriptive analysis, signal detection using four disproportionality methods (ROR, PRR, EBGM, and BCPNN), signal prioritization, and time-to-onset modeling.

#### Descriptive analysis

Descriptive statistics were applied to the collected reports to categorize the adverse events(AEs) reports of givosiran by gender, age, reported countries, outcome, reporting year and reporters category.

#### Pharmacovigilance disproportionality analysis

This research report strictly follows the READUS-PV checklist, which is now one of the recommended reporting frameworks for pharmacovigilance signal-detection studies [13]. Signal strength of the four analytical indicators (ROR, PRR, EBGM, and BCPNN) is positively correlated with the statistical association between givosiran and AEs [14].

These algorithms were used to determine the reporting risk for quantifying drug–AEs associations based on the two-by-two contingency table that captures drug presence or absence and event occurrence in case reports [15]. Table 1 show the two-by-two contingency Tables [16, 17].

**Table 1:** The two-by-two contingency table for disproportionality analysis

	Target adverse reaction	All other adverse events	Total
Givosiran	A	B	A + B
All other drugs	C	D	C + D
Total	A + C	B + D	A + B+C + D=N

According to the ROR, a positive risk of adverse event reporting was defined as the case count ≥ 3 and lower limit of 95% confidence interval (CI) > 1. For the PRR, a positive risk of adverse event reporting was defined as the case count ≥ 3.0 and PRR value ≥ 2.0, accompanied by an associated χ2 value ≥ 4. In the BCPNN algorithm, the risk of adverse event reporting was assessed by the IC_025_ index, which is the lower limit of the 95% bilateral confidence interval for IC. IC_025_ value > 0 indicates a positive risk of adverse event reporting. For the EBGM: The lower limit of the 95% confidence interval for the Empirical Bayes Geometric Mean (EBGM_05_) > 2 indicates a positive risk of adverse event reporting [18].

Each algorithm possesses distinct advantages and disadvantages in terms of its applicability across different scenarios and potential for implementation [19]. Comparative analyses of specificity have revealed that no single algorithm universally outperforms others [20]. The adverse events in this study were classified as related to givosiran if they were identified by all of the above criteria for the four indices. In addition, a sensitivity analysis was conducted after excluding PTs potentially related to the underlying manifestations of acute hepatic porphyria to evaluate the robustness of the detected signals.

#### Prioritization of relevant disproportionality signals

To enhance the clinical interpretability of statistically detected signals, a semiquantitative prioritization framework was applied. The present framework was derived from well‑recognized pharmacovigilance protocols in previous studies, and further adjusted to match the analytical strategy adopted herein [21].

The prioritization incorporated four dimensions: clinical relevance, reporting rate, signal stability, and reported case fatality rate. Clinical relevance was assessed using the lists of Important Medical Events (IME) and Designated Medical Events (DME), as defined by the European Medicines Agency. The reporting rate was calculated as the proportion of a specific adverse event among all reported events for the drug, and categorized as very common (≥ 10%), common (1–10%), or uncommon (< 1%). Signal stability was defined as the consistency of positive signals across the four disproportionality algorithms used in this study (ROR, PRR, EBGM, and BCPNN). Full consistency across all four methods (4/4) was assigned the highest score, consistency across two or three methods (2/4–3/4) was assigned an intermediate score, and consistency across two or fewer methods (≤ 1/4) was assigned the lowest score. This modification was introduced to ensure alignment between the signal detection criteria and the prioritization framework. The reported case fatality rate was calculated as the proportion of reports with a fatal outcome among all reports for each adverse event. The highest score was assigned only when the fatality rate exceeded 50%.

Each criterion was assigned a score ranging from 0 to 2, resulting in a total score ranging from 0 to 8. Based on the total score, adverse events were classified into three priority levels: low priority (0–2), moderate priority (3–5), and high priority (6–8) (detailed in Table 2).

**Table 2:** Criteria and relevant scores to prioritize AEs emerged from disproportionality analysis

Criterium	2 points	1 point	0 point
Reporting rate	> 10%	1–10%	0–1%
Signal stability	4/4	2/4–3/4	≤ 1/4
Reported case fatality rate	> 50%	25–50%	< 25%
Clinical relevance	DME	IME	None

DME: Designated Medical Events; IME: Important Medical Events

#### Time to onset (TTO) analysis

As defined in this study, the TTO of a givosiran AE is the time interval between the EVENT_DT date recorded in the DEMO file and the START_DT date recorded in the THER file.Cases lacking complete date information were omitted. To characterize the TTO profile of givosiran, we evaluated four candidate parametric distributions: Log-Normal, Weibull, Gamma, and Exponential. The optimal distribution for fitting the TTO data was determined through goodness-of-fit (GOF) tests. Specifically, the Akaike Information Criterion (AIC) and − 2*lg L(β) were utilized as the primary selection criteria. By leveraging its scale (α) and shape (β) parameters, the model allows for a precise identification of risk patterns. In this study, the shape parameter (β) served as the central metric, as it provides critical insights into the AE risk incidence throughout the observation period. If β is less than 1, and its 95% confidence interval (CI) is also below 1, the risk of adverse effects is considered to diminish over time, reflecting an early failure-type curve. In contrast, if β is close to 1 and its 95% CI encompasses 1, the risk is considered constant over time, corresponding to a random failure-type curve. Finally, if β exceeds 1 and its 95% CI does not include 1, the hazard is interpreted as rising over time, indicating a wear-out failure-type curve.

## Results

### Results of pharmacovigilance analysis

#### Data extraction results

**Fig. 1:**
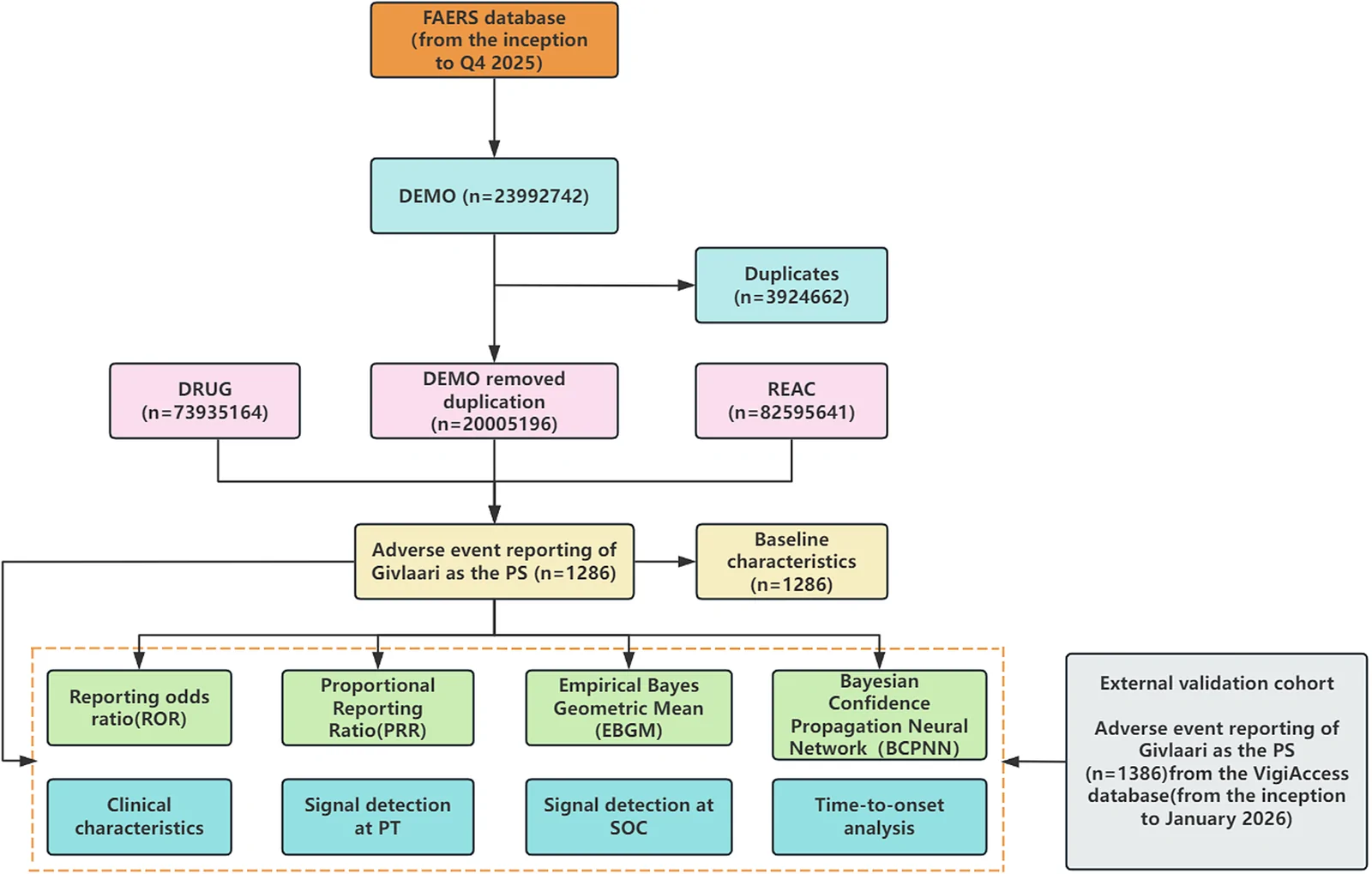
Flowchart of data extraction and processing

A total of 23,992,742 AEs reports were extracted from the FAERS database. Following rigorous data cleaning and deduplication, 1,286 reports in which givosiran was designated as the Primary Suspect (PS) were identified for the final analysis (Fig. 1).

#### Descriptive overview of cases

Among givosiran reports with available gender and age information, female patients and individuals aged 18–44 years constituted the largest proportion. However, the majority of reports lacked demographic information, limiting the interpretability of these findings. The Top five reporting countries were the United States (1109 cases, 86.2%), followed by Germany (19 cases, 1.5%), Canada (18 cases, 1.4%), Italy (17 cases, 1.3%), Great Britain (14 cases, 1.1%). The largest number of reported years is 2024(304 cases, 23.6%). The main reporters were consumer(753 cases, 58.6%), and the main outcome was hospitalization(611 cases, 47.5%). Detailed demographic statistics are presented in Table 3.

**Table 3:** Clinical characteristics of givosiran-associated AEs in the FAERS database

Clinical characteristics	Givosiran(*N* = 1286)
Gender	
Male	3 (0.2%)
Female	27 (2.1%)
Missing	1256 (97.7%)
Age (years old)	
0 ~ 17	1 (0.1%)
18 ~ 44	24 (1.8%)
45 ~ 64	18 (1.4%)
≥ 65	5 (0.4%)
Missing	1238 (96.3%)
Reported Countries(Top 5)	
United States	1109 (86.2%)
Germany	19 (1.5%)
Canada	18 (1.4%)
Italy	17 (1.3%)
Great Britain	14 (1.1%)
Reported year	
2020	70 (5.4%)
2021	123 (9.6%)
2022	221 (17.2%)
2023	268 (20.8%)
2024	304 (23.6%)
2025	300 (23.3%)
Reporters	
Consumer	753 (58.6%)
Physician	250 (19.4%)
Health Professional	199 (15.5%)
Pharmacist	14 (1.1%)
Missing	70 (5.4%)
Outcome	
Hospitalization	611 (47.5%)
Death	35 (2.7%)
Disability	15 (1.2%)
Life-Threatening	15 (1.2%)
Other	610 (47.4%)

#### Disproportionality analysis

**Fig. 2:**
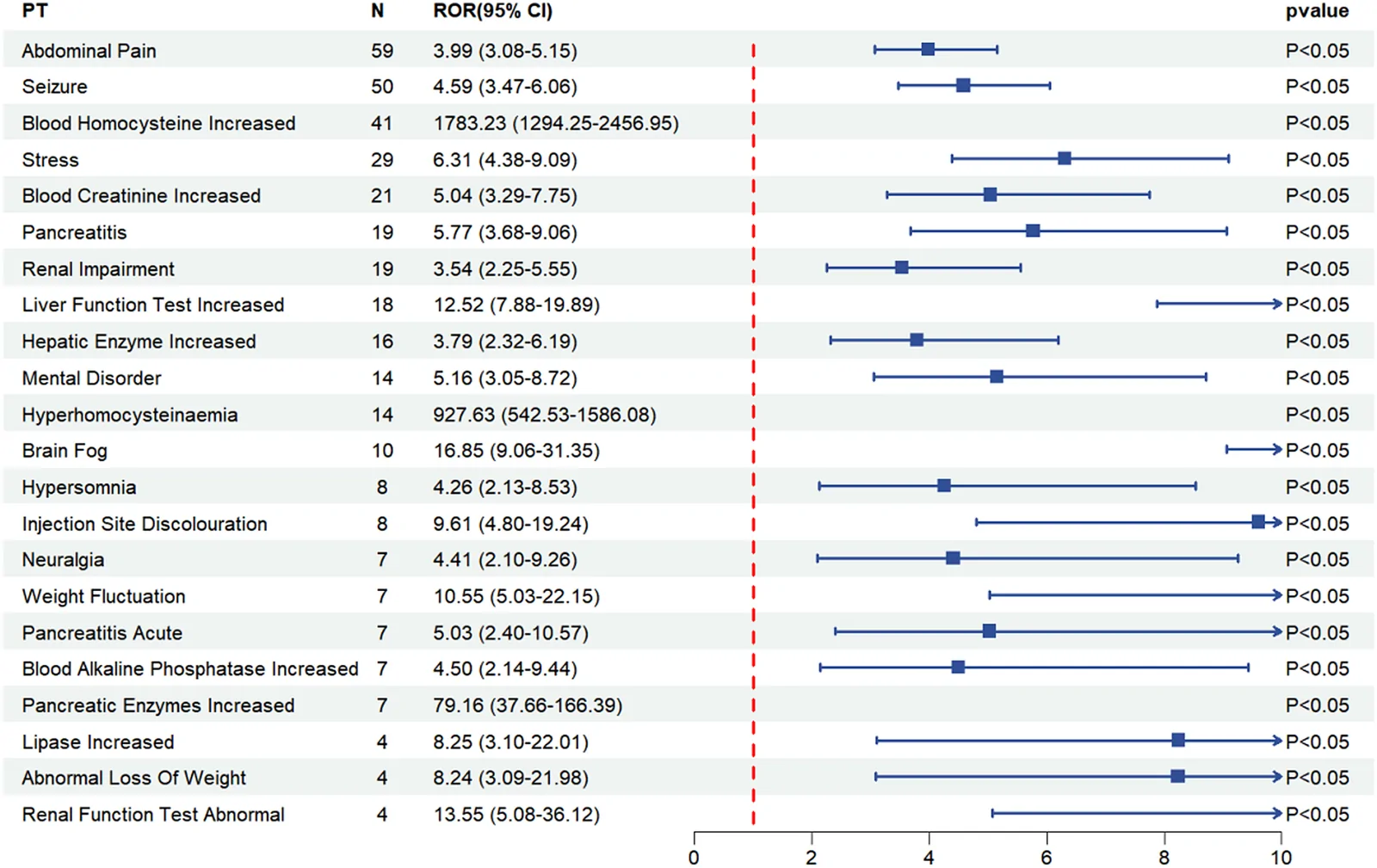
Distribution of adverse events at the PT level ranked by frequency

**Fig. 3:**
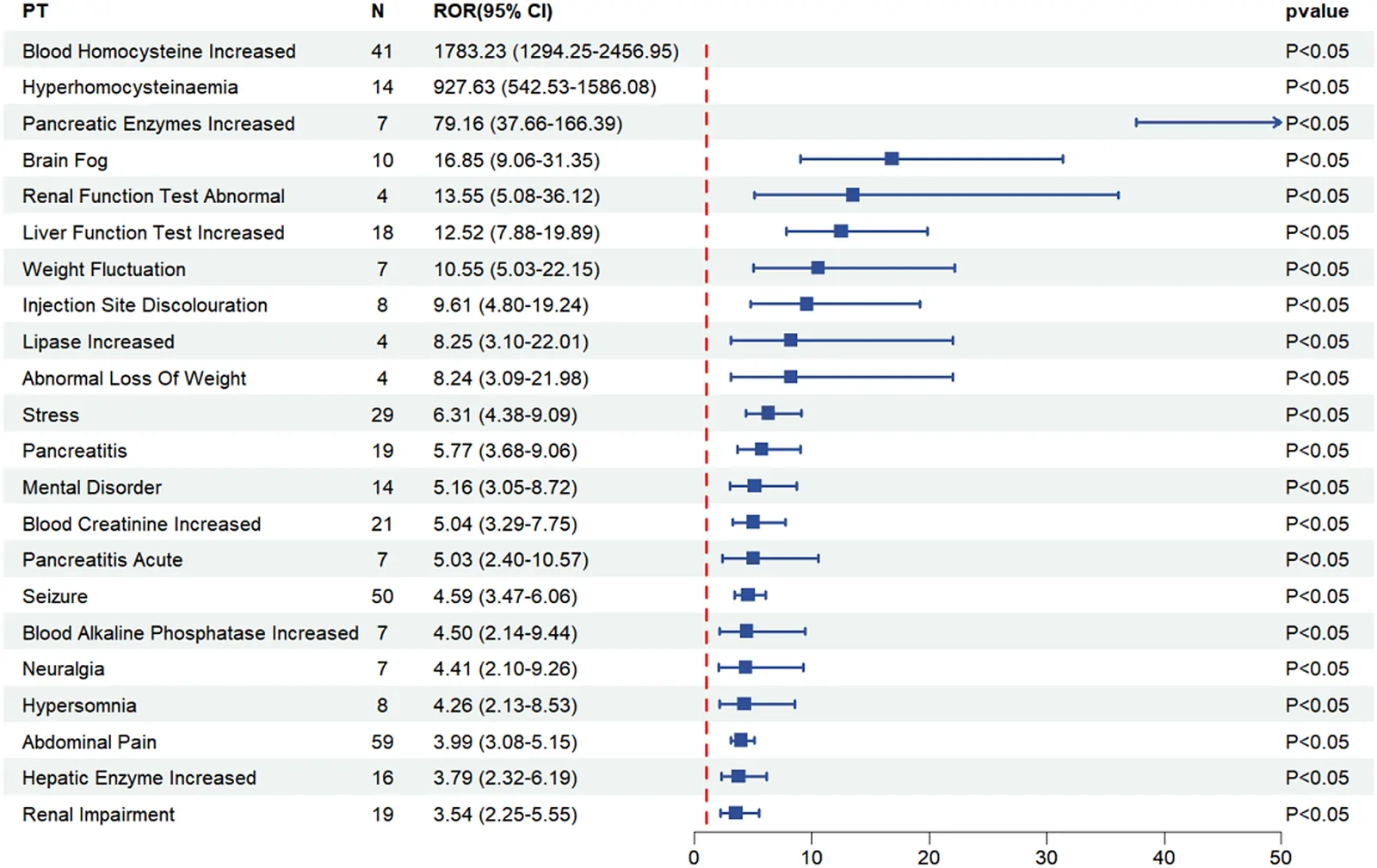
Distribution of adverse events at the PT level ranked by ROR

**Fig. 4:**
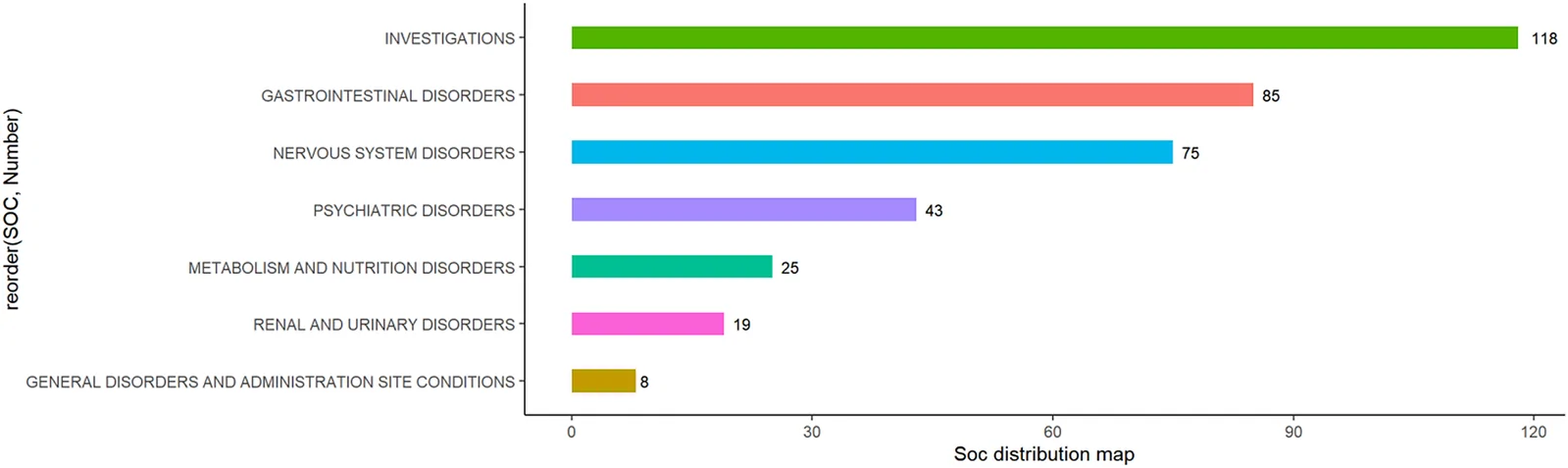
SOC distribution of givosiran-associated adverse events in the FAERS database

Table 4 shows that after evaluating 1,286 reports related to givosiran, a total of 373 reported reports were detected, disproportionality analysis identified 22 significant PT signals in FAERS. Among these, nine new PTs not listed in the drug’s package insert were discovered, including abdominal pain, seizure, stress, mental disorder, brain fog, hypersomnia, neuralgia, weight fluctuation and abnormal loss of weight. Ranked by the frequency (Fig. 2), the top 5 PTs include abdominal pain (59cases), seizure (50cases), blood homocysteine increased (41cases), stress (29cases), and blood creatinine increased (21cases). Ranked by ROR (Fig. 3), the top 5 PTs include blood homocysteine increased (ROR 1783.23, 95%CI 1294.25–2456.95), hyperhomocysteinaemia (ROR 927.63, 95%CI 542.53–1586.08), pancreatic enzymes increased (ROR 79.16, 95%CI 37.66–166.39), brain fog (ROR 16.85, 95%CI 9.06–31.35), and renal function test abnormal (ROR 13.55, 95%CI 5.08–36.12). Figure 4 illustrates the distribution of givosiran-associated positive signals at the SOC level, ranked by reporting frequency. Our analysis identified that the AEs profile for givosiran encompassed 7 distinct SOCs. Including investigations gastrointestinal disorders (118cases, 31.63%), nervous system disorders(85cases, 22.79%), psychiatric disorders(75cases, 20.11%), metabolism and nutrition disorders(43cases, 11.53%), renal and urinary disorders(25cases, 6.70%), general disorders(19cases, 5.09%) and administration site conditions(8cases, 2.15%). Detailed disproportionality analysis results for givosiran-associated adverse events in the FAERS database are given in Supplementary files 1.

**Table 4:** Detection of positive signals associated with givosiran in the FAERS database

PT	*N*	ROR (95% CI)	PRR (χ2)	EBGM(EBGM_05_)	IC (IC_025_)	Package insert records the risk
Abdominal pain	59	3.99 (3.08–5.15)	3.94 (130.02)	3.94 (3.05)	1.98 (1.53)	No
Seizure	50	4.59 (3.47–6.06)	4.54 (138.52)	4.54 (3.44)	2.18 (1.68)	No
Blood homocysteine increased	41	1783.23 (1294.25–2456.95)	1765.06 (66564.45)	1625.43 (1179.72)	10.67 (4.89)	Yes
Stress	29	6.31 (4.38–9.09)	6.27 (128.55)	6.27 (4.35)	2.65 (1.89)	No
Blood creatinine increased	21	5.04 (3.29–7.75)	5.02 (67.73)	5.02 (3.27)	2.33 (1.47)	Yes
Pancreatitis	19	5.77 (3.68–9.06)	5.75 (74.54)	5.75 (3.66)	2.52 (1.57)	Yes
Renal impairment	19	3.54 (2.25–5.55)	3.53 (34.44)	3.53 (2.25)	1.82 (1)	Yes
Liver function test increased	18	12.52 (7.88–19.89)	12.47 (189.81)	12.46 (7.84)	3.64 (2.29)	Yes
Hepatic enzyme increased	16	3.79 (2.32–6.19)	3.77 (32.66)	3.77 (2.31)	1.92 (1)	Yes
Mental disorder	14	5.16 (3.05–8.72)	5.15 (46.8)	5.15 (3.04)	2.36 (1.26)	No
Hyperhomocysteinaemia	14	927.63 (542.53–1586.08)	924.4 (12357.43)	884.63 (517.38)	9.79 (3.12)	Yes
Brain fog	10	16.85 (9.06–31.35)	16.81 (148.62)	16.8 (9.03)	4.07 (1.91)	No
Hypersomnia	8	4.26 (2.13–8.53)	4.26 (19.94)	4.26 (2.13)	2.09 (0.68)	No
Injection site discolouration	8	9.61 (4.8–19.24)	9.6 (61.58)	9.59 (4.79)	3.26 (1.33)	Yes
Neuralgia	7	4.41 (2.1–9.26)	4.41 (18.43)	4.4 (2.1)	2.14 (0.61)	No
Weight fluctuation	7	10.55 (5.03–22.15)	10.53 (60.39)	10.53 (5.02)	3.4 (1.24)	No
Pancreatitis acute	7	5.03 (2.4–10.57)	5.03 (22.58)	5.03 (2.39)	2.33 (0.72)	Yes
Blood alkaline phosphatase increased	7	4.5 (2.14–9.44)	4.49 (19)	4.49 (2.14)	2.17 (0.62)	Yes
Pancreatic enzymes increased	7	79.16 (37.66–166.39)	79.02 (537.2)	78.72 (37.45)	6.3 (1.85)	Yes
Lipase increased	4	8.25 (3.1–22.01)	8.25 (25.47)	8.24 (3.09)	3.04 (0.46)	Yes
Abnormal loss of weight	4	8.24 (3.09–21.98)	8.24 (25.42)	8.23 (3.09)	3.04 (0.46)	No
Renal function test abnormal	4	13.55 (5.08–36.12)	13.53 (46.41)	13.53 (5.07)	3.76 (0.66)	Yes

#### Clinical prioritization of relevant disproportionality signals

The clinical priority analysis for givosiran in the FAERS database, detailed in Table 5, synthesized four critical dimensions: clinical relevance (including IME/DME status), reporting rate, fatality rate, and signal stability. Pancreatitis acute and pancreatitis are classified as DME, while seizure and renal impairment are classified as IME. Blood homocysteine increased, pancreatitis acute, seizure, pancreatitis, Abdominal pain, and renal impairment are designated as moderate priority; all other PTs are designated as low priority.

**Table 5:** Clinical priority analysis of givosiran-associated PTs in the FAERS database

PT	Clinical relevance	Reporting rate	Fatality rate (%)	Total score	Relevant prioritization
Blood homocysteine increased	-	1.02%	0	3	Moderate priority
Hyperhomocysteinaemia	-	0.35%	0	2	Low priority
Pancreatic enzymes increased	-	0.17%	0	2	Low priority
Renal function test abnormal	-	0.10%	0	2	Low priority
Liver function test increased	-	0.45%	0	2	Low priority
Brain fog	-	0.25%	0	2	Low priority
Injection site discolouration	-	0.20%	0	2	Low priority
Weight fluctuation	-	0.17%	0	2	Low priority
Abnormal loss of weight	-	0.10%	0	2	Low priority
Lipase increased	-	0.10%	0	2	Low priority
Stress	-	0.73%	0	2	Low priority
Pancreatitis acute	DME	0.17%	14.29%	4	Moderate priority
Seizure	IME	1.26%	0	4	Moderate priority
Pancreatitis	DME	0.47%	0	4	Moderate priority
Blood creatinine increased	-	0.52%	0	2	Low priority
Neuralgia	-	0.17%	0	2	Low priority
Mental disorder	-	0.35%	0	2	Low priority
Blood alkaline phosphatase increased	-	0.17%	0	2	Low priority
Hypersomnia	-	0.20%	0	2	Low priority
Abdominal pain	-	1.49%	0	3	Moderate priority
Hepatic enzyme increased	-	0.40%	0	2	Low priority
Renal impairment	IME	0.47%	0	3	Moderate priority

#### TTO analysis of AEs associated with givosiran

**Fig. 5:**
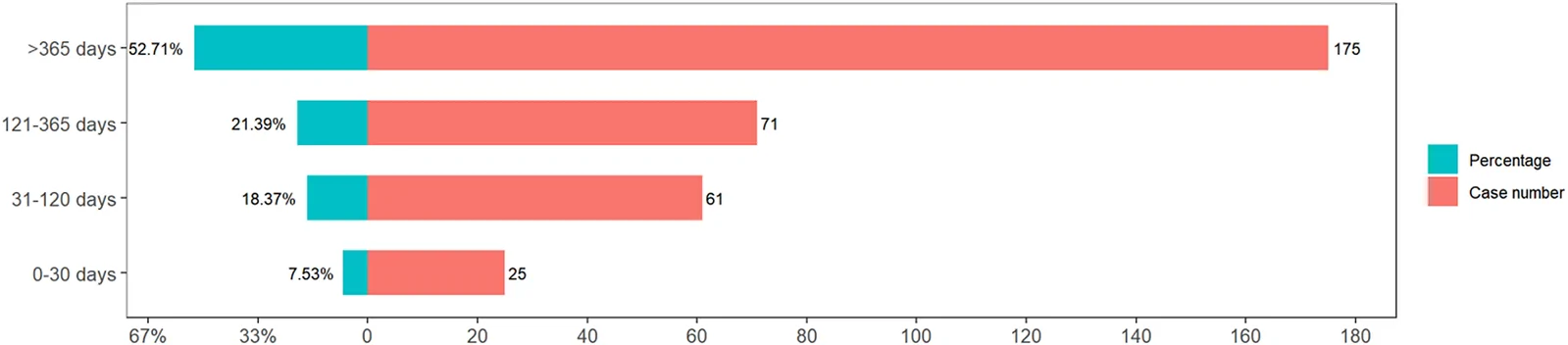
Time-to-onset distribution of givosiran-associated adverse events

A total of 332 adverse event reports containing the onset time of givosiran were retrieved from the FAERS database. Analysis shows that the majority of AEs (175 cases, 52.71%) occurred 365 days after administration. The mean time to onset of AEs was 544.36 days, with a minimum and maximum range of 1 to 994 days, as shown in Fig. 5; Table 7.

**Fig. 6:**
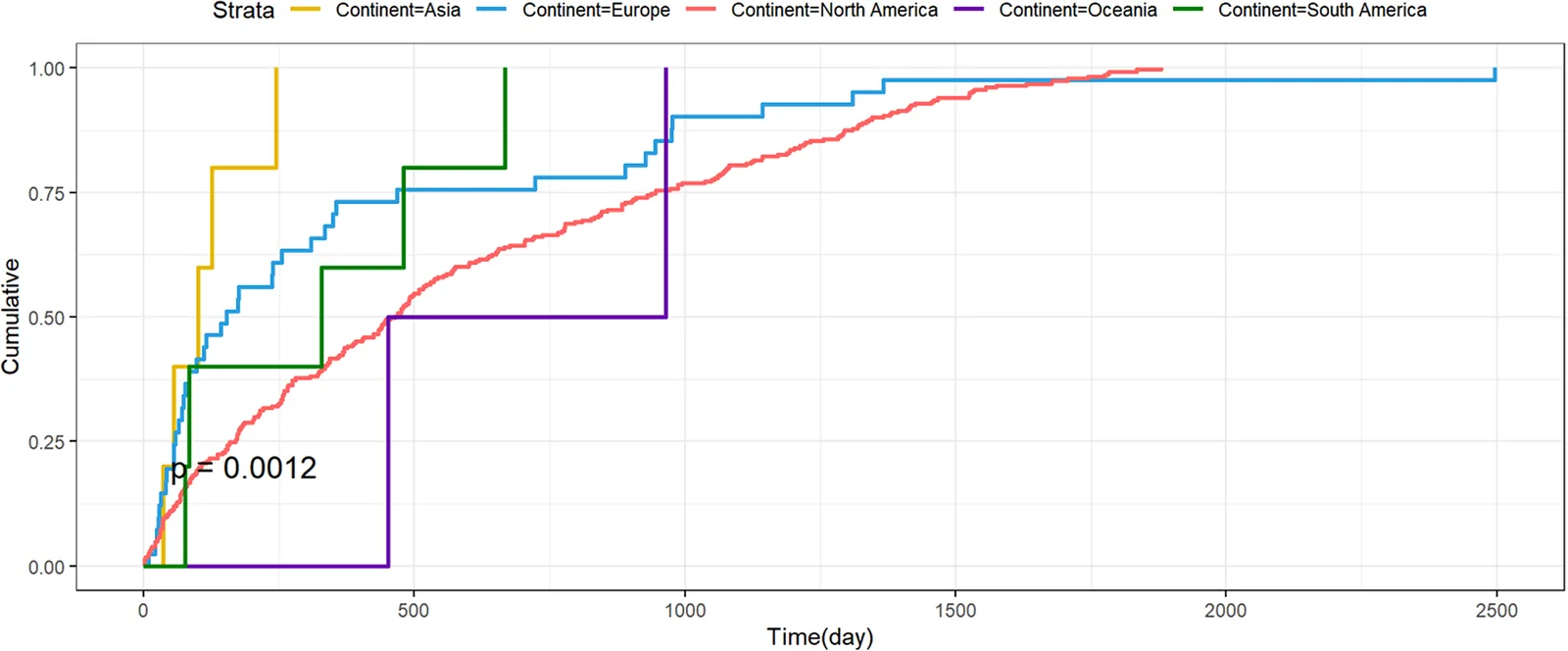
Subgroup analysis of TTO by continent

Survival analysis indicated a statistically significant difference in time-to-onset of AEs associated with givosiran between Continent recipients (*p* = 0.0012 < 0.05), as shown in Fig. 6.

The goodness-of-fit test results (Table 6) show that the Weibull distribution model has the smallest AICc (4828.356) and − 2*lg L(β) (4828.32), making it the best model for describing givosiran-related adverse event TTO data. The shape parameter (β) for the onset time of AEs associated with givosiran were estimated to be 1.01, with a 95% CI of 0.73–1.26, suggesting the risk was considered constant over time, corresponding to a random failure-type curve (see Table 7).

**Table 6:** Performance testing of goodness-of-fit among four parametric distribution models

Model	AICc	BIC	−2*lg L(β)
Log-Normal	4845.753	4857.327	4845.717
Weibull	4828.356	4839.93	4828.32
Gamma	4843.812	4855.385	4843.775
Exponential	4846.955	4852.74	4846.943

*The minimum AICc and − 2*lg L(β) of Model indicate a good fit

**Table 7:** Weibull distribution analysis of givosiran-associated AEs in the FAERS database

Drug	Case(*n*)	Overall deseription TTO (days)	Weibull distribution test				
		Media(Min-Max)	scale parameter		Shape parameter		Failure type
			α	95% CI	β	95% CI	
Givosiran	332	544.36(1-994)	296.21	193.06-399.36	1.01	0.73–1.26	Random failure-type

#### Sensitivity analysis

To reduce potential confounding by indication, a sensitivity analysis was conducted after excluding several PTs that may represent underlying manifestations or complications of acute hepatic porphyria, including renal function test abnormal, seizure, blood creatinine increased, neuralgia, mental disorder, abdominal pain, and renal impairment.

Following exclusion of these PTs, disproportionality analysis continued to identify multiple statistically significant signals associated with givosiran (Table 8). The strongest signals remained related to homocysteine metabolism, including blood homocysteine increased (ROR 1846.11, 95% CI 1339.80–2543.76) and hyperhomocysteinaemia (ROR 960.04, 95% CI 561.46–1641.57). Pancreatic-related adverse events, including pancreatitis, acute pancreatitis, pancreatic enzymes increased, and lipase increased, also remained significant. In addition, hepatic-related signals such as liver function test increased and hepatic enzyme increased continued to show positive disproportionality. Several non-labeled signals, including brain fog, hypersomnia, stress, weight fluctuation, and abnormal loss of weight, also persisted after sensitivity analysis.

**Table 8:** Positive signals associated with givosiran after excluding 7 PTs related to AHP-related symptoms

PT	*N*	ROR (95% CI)	PRR (χ2)	EBGM(EBGM_05_)	IC (IC_025_)	Package insert records the risk
Blood homocysteine increased	41	1846.11 (1339.80–2543.76)	1826.45 (68882.44)	1681.96 (1286.26)	10.71 (9.04)	Yes
Stress	29	6.52 (4.536–9.41)	6.48 (134.72)	6.48 (4.77)	2.69 (1.03)	No
Pancreatitis	19	5.97 (3.80–9.37)	5.94 (78.23)	5.94 (4.07)	2.57 (0.90)	Yes
Liver function test increased	18	12.95 (8.15–20.59)	12.90 (197.57)	12.89 (8.75)	3.68 (2.02)	Yes
Hepatic enzyme increased	16	3.91 (2.39–6.40)	3.90 (34.61)	3.90 (2.58)	1.96 (0.29)	Yes
Hyperhomocysteinaemia	14	960.04 (561.46–1641.57)	956.55 (12788.20)	915.39 (584.35)	9.83 (8.16)	Yes
Brain fog	10	17.44 (9.37–32.44)	17.39 (154.44)	17.38 (10.34)	4.11 (2.45)	No
Hypersomnia	8	4.41 (2.20–8.83)	4.40 (21.06)	4.40 (2.46)	2.13 (0.47)	No
Injection site discolouration	8	9.94 (4.97–19.91)	9.92 (64.22)	9.92 (5.55)	3.31 (1.64)	Yes
Weight fluctuation	7	10.91 (5.20–22.92)	10.90 (62.92)	10.89 (5.85)	3.44 (1.77)	No
Pancreatitis acute	7	5.20 (2.48–10.93)	5.20 (23.75)	5.19 (2.79)	2.37 (0.71)	Yes
Blood alkaline phosphatase increased	7	4.65 (2.21–9.76)	4.64 (20.03)	4.64 (2.49)	2.21 (0.54)	Yes
Pancreatic enzymes increased	7	81.92 (38.97–172.19)	81.77 (556.36)	81.46 (43.75)	6.34 (4.68)	Yes
Lipase increased	4	8.54 (3.206–22.77)	8.53 (26.59)	8.53 (3.75)	3.09 (1.42)	Yes
Abnormal loss of weight	4	8.52 (3.19–22.74)	8.52 (26.54)	8.51 (3.74)	3.09 (1.42)	No

#### External validation in VigiAccess database

**Fig. 7:**
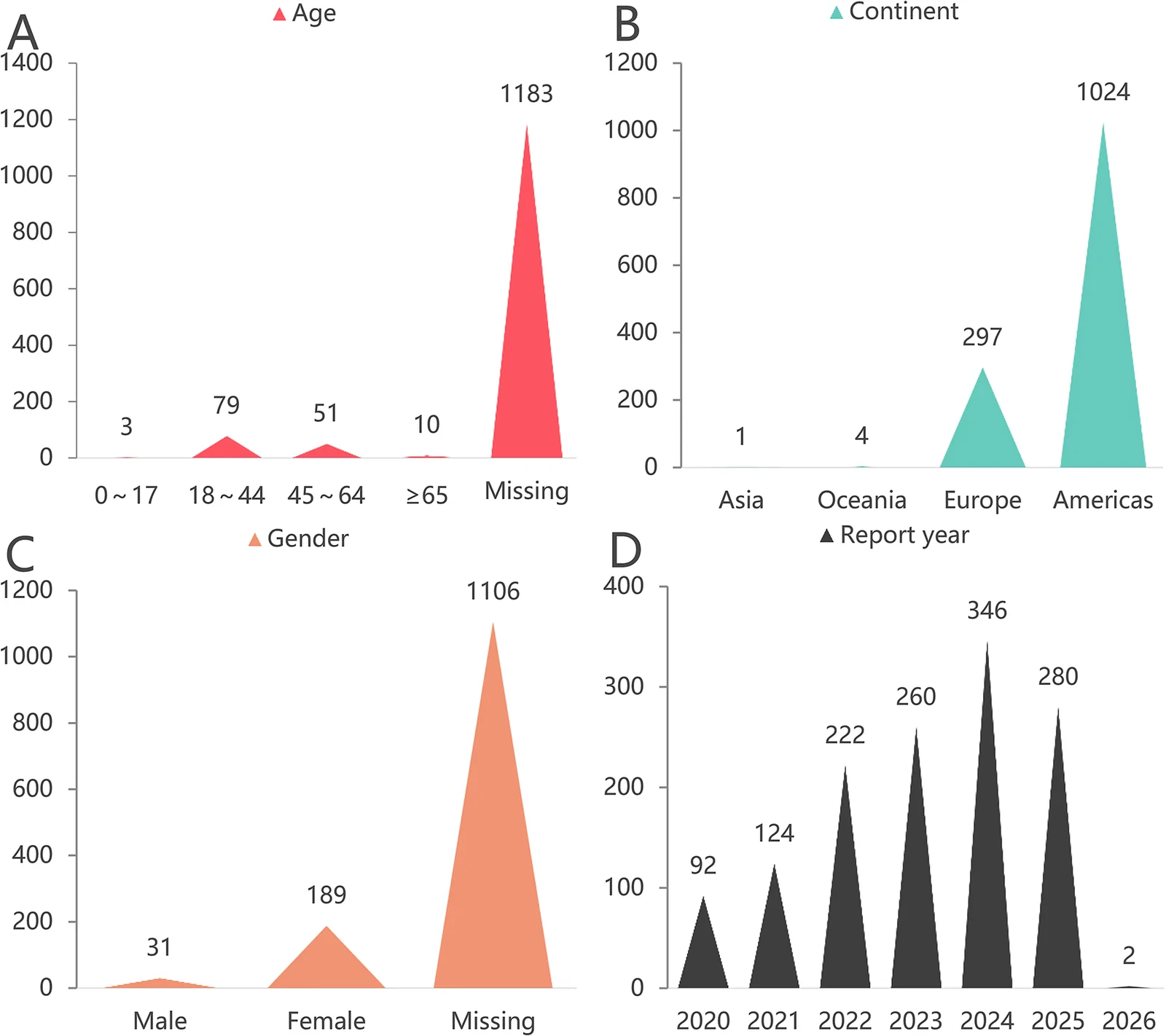
Statistical distribution of AEs by age, continent, gender, and report year (**A**, **B**, **C**, and **D**)

The VigiAccess database was employed as a secondary data source to corroborate the initial findings. A limited total of 1,326 reports of AEs associated with givosiran were collected. When all four detection methods were positive simultaneously, a total of 35 signals were detected, including 13 newly discovered adverse events not included in the drug’s package insert. In the FAERS database, only brain fog was not detected in the VigiAccess database, all other PTs were detected (Table 9). Demographic features (age, continent, gender and report year) are illustrated in Fig. 7A–D. Detailed disproportionality analysis results for givosiran-associated adverse events in the VigiAccess database are given in Supplementary files 2.

**Table 9:** Detection of positive signals associated with givosiran in the VigiAccess database

PT	*N*	ROR (95% CI)	PRR (χ2)	EBGM(EBGM_05_)	IC (IC_025_)	Package insert records the risk
Abdominal pain	77	7.31 (5.83–9.17)	7.16 (409.59)	7.16 (5.93)	2.84 (1.17)	No
Blood homocysteine increased	62	2270.56 (1737.43–2967.29)	2228.14 (121405.8)	1960.02 (1566.78)	10.94 (9.27)	Yes
Seizure	46	5.71 (4.27–7.64)	5.65 (176.28)	5.65 (4.43)	2.5 (0.83)	No
Hyperhomocysteinaemia	38	2870.64 (2030.51–4058.38)	2837.76 (91763.97)	2416.68 (1808.82)	11.24 (9.56)	Yes
Blood creatinine increased	31	10.21 (7.17–14.55)	10.13 (255.08)	10.12 (7.53)	3.34 (1.67)	Yes
Stress	30	7.98 (5.57–11.44)	7.92 (181.46)	7.91 (5.86)	2.98 (1.32)	No
Renal impairment	24	6.44 (4.31–9.63)	6.41 (109.55)	6.4 (4.58)	2.68 (1.01)	Yes
Hepatic enzyme increased	21	5.83 (3.80–8.96)	5.8 (83.53)	5.8 (4.05)	2.54 (0.87)	Yes
Pancreatitis	17	6.52 (4.05–10.50)	6.49 (78.95)	6.49 (4.35)	2.7 (1.03)	Yes
Liver function test increased	16	16.65 (10.19–27.22)	16.58 (234.05)	16.56 (10.98)	4.05 (2.38)	Yes
Lipase increased	15	34.29 (20.64–56.98)	34.14 (481.65)	34.07 (22.28)	5.09 (3.42)	Yes
Injection site reaction	13	3.59 (2.08–6.20)	3.58 (24.24)	3.58 (2.27)	1.84 (0.17)	Yes
Alanine aminotransferase increased	12	3.74 (2.12–6.59)	3.73 (23.99)	3.73 (2.32)	1.9 (0.23)	Yes
Mental disorder	12	4.88 (2.77–8.60)	4.86 (36.85)	4.86 (3.03)	2.28 (0.62)	No
Transaminases increased	12	101.22 (57.32–178.73)	100.86 (1179.13)	100.24 (62.29)	6.65 (4.98)	Yes
Injection site discolouration	10	13.91 (7.48–25.89)	13.87 (119.37)	13.86 (8.24)	3.79 (2.13)	Yes
Blood alkaline phosphatase increased	9	6.63 (3.44–12.75)	6.61 (42.85)	6.61 (3.82)	2.72 (1.06)	Yes
Glomerular filtration rate decreased	9	16.17 (8.40–31.11)	16.13 (127.6)	16.11 (9.32)	4.01 (2.34)	Yes
Hypersomnia	9	5.77 (3.00–11.10)	5.76 (35.39)	5.76 (3.33)	2.53 (0.86)	No
Pancreatic enzymes increased	9	108.82 (56.45–209.79)	108.53 (952.54)	107.82 (62.25)	6.75 (5.08)	Yes
Hepatic function abnormal	7	3.6 (1.72–7.56)	3.6 (13.13)	3.6 (1.93)	1.85 (0.18)	Yes
Pancreatitis acute	7	6.53 (3.11–13.70)	6.51 (32.68)	6.51 (3.50)	2.7 (1.04)	Yes
Amylase increased	6	20.01 (8.98–44.58)	19.97 (108)	19.95 (10.20)	4.32 (2.65)	Yes
Neuralgia	6	4.3 (1.93–9.58)	4.29 (15.16)	4.29 (2.20)	2.1 (0.44)	No
Presyncope	6	3.97 (1.78–8.84)	3.96 (13.29)	3.96 (2.03)	1.99 (0.32)	No
Weight fluctuation	6	10.81 (4.85–24.09)	10.79 (53.28)	10.79 (5.52)	3.43 (1.76)	No
Renal function test abnormal	5	23.22 (9.65–55.85)	23.18 (105.99)	23.15 (11.11)	4.53 (2.87)	Yes
Abnormal loss of weight	4	11.67 (4.38–31.12)	11.66 (38.94)	11.65 (5.13)	3.54 (1.87)	No
Polyneuropathy	4	7.05 (2.64–18.80)	7.04 (20.73)	7.04 (3.10)	2.82 (1.15)	No
Renal pain	4	7.21 (2.70–19.23)	7.21 (21.37)	7.2 (3.17)	2.85 (1.18)	Yes
Blood glucose fluctuation	3	5.1 (1.64–15.83)	5.1 (9.88)	5.1 (1.98)	2.35 (0.68)	No
Energy increased	3	9.07 (2.92–28.15)	9.06 (21.51)	9.06 (3.51)	3.18 (1.51)	No
Hypotonia	3	5.9 (1.90–18.31)	5.9 (12.20)	5.9 (2.29)	2.56 (0.89)	No
Injection site discomfort	3	5.7 (1.84–17.68)	5.69 (11.60)	5.69 (2.21)	2.51 (0.84)	Yes
Secondary adrenocortical insufficiency	3	35.92 (11.56–111.56)	35.88 (101.52)	35.81 (13.87)	5.16 (3.49)	Yes

## Discussion

### Overview of key findings

This study provides a comprehensive pharmacovigilance evaluation of givosiran using two independent databases. Several adverse events were consistently identified across analyses, including both established and potential emerging safety signals. The overall consistency between the FAERS and VigiAccess databases supports the robustness of the findings. To improve interpretability, the following sections discuss established safety signals and potential emerging signals separately, followed by signal prioritization and time-to-onset analysis.

### Major positive signals associated with givosiran

Among the detected signals, increased blood homocysteine and hyperhomocysteinaemia demonstrated exceptionally strong disproportionality values in both databases. These findings are consistent with emerging clinical evidence indicating that givosiran therapy has been associated with elevated plasma homocysteine levels in previous studies. Givosiran exerts its therapeutic effect by silencing hepatic ALAS1 expression, thereby reducing the accumulation of neurotoxic heme precursors such as δ-aminolevulinic acid (ALA) and porphobilinogen. However, ALAS1 inhibition may also interfere with hepatic heme-dependent metabolic pathways, including those involved in vitamin B6–dependent transsulfuration and homocysteine metabolism [22, 23]. Elevated homocysteine levels are clinically important because they have been associated with an increased risk of cardiovascular complications, neurotoxicity, and cognitive impairment. Therefore, routine monitoring of homocysteine levels and consideration of vitamin supplementation may be warranted in patients receiving long-term givosiran therapy.

Renal-related signals, including renal impairment and increased blood creatinine, were consistently observed in both databases. Kidney involvement is a recognized complication of acute hepatic porphyria itself, particularly in patients with recurrent attacks [24]. Therefore, the observed signals may reflect, at least in part, the underlying disease process or confounding by indication. Although a statistical association between givosiran use and renal-related events was detected, these findings should be interpreted cautiously and cannot establish a causal relationship. Further studies are warranted to clarify whether these signals represent drug-related effects or disease-related manifestations.

Pancreatitis and acute pancreatitis were identified as moderate-priority signals in the clinical prioritization analysis. Although pancreatitis has been reported in previous clinical observations, the mechanisms underlying this association remain unclear [6]. Elevated pancreatic enzyme-related signals were also observed in the present analysis; however, whether these findings represent early manifestations of pancreatic involvement or reporting patterns associated with clinically recognized pancreatitis remains unclear. Clinicians should therefore carefully differentiate between abdominal pain caused by AHP attacks and that resulting from potential pancreatic complications during givosiran therapy.

### Established and potential emerging positive signals

Several adverse events identified in this study are consistent with previously recognized safety concerns associated with givosiran, including hyperhomocysteinaemia, renal impairment, and pancreatitis. These findings are in line with clinical trial data and post-marketing observations, supporting the robustness of our analytical approach.

One notable finding of this study was the identification of eight potential adverse events that were consistently detected in both FAERS and VigiAccess databases but are not currently included in the drug labeling. These signals include abdominal pain, seizure, stress, mental disorder, hypersomnia, neuralgia, weight fluctuation, and abnormal loss of weight.

Certain signals may inherently reflect the underlying clinical manifestations of AHP rather than drug-induced effects. For instance, abdominal pain—a hallmark of acute attacks—likely represents confounding by indication. Similarly, while neurological and psychiatric PTs (such as seizure, mental disorder, and hypersomnia) were consistently identified across both databases, these findings warrant cautious interpretation. Although the neurotoxic effects of porphyrin precursors like δ-aminolevulinic acid (ALA) provide biological plausibility for such events [25], which has been shown to interfere with neurotransmitter systems including γ-aminobutyric acid (GABA), they remain hypothesis-generating given their symptomatic overlap with AHP itself. In addition, previous studies have reported potential associations between elevated homocysteine levels and neurological dysfunction; however, the clinical relevance of these findings in patients receiving givosiran remains uncertain.

Weight fluctuation and abnormal weight loss may be secondary to gastrointestinal symptoms, metabolic changes, or hepatic dysfunction associated with givosiran treatment. Although causal relationships cannot be confirmed through pharmacovigilance data alone, these findings highlight potential safety concerns that should be further explored in prospective clinical studies.

### Clinical prioritization of positive signals

We applied a adapted prioritization framework improves consistency between signal detection and clinical interpretation, and may be applicable to future pharmacovigilance studies using multiple algorithms, incorporating reporting rate, signal stability, fatality rate, and clinical relevance. Based on this analysis, acute pancreatitis, seizure, abdominal pain, renal impairment, and blood homocysteine increased were classified as moderate-priority signals. These findings suggest that clinicians should pay particular attention to neurological symptoms, pancreatic complications, and metabolic abnormalities during givosiran therapy. The integration of clinical prioritization into pharmacovigilance analyses may help translate statistical signals into clinically meaningful safety information, thereby supporting risk management and clinical decision-making.

### TTO and risk patterns

Time-to-onset analysis revealed a mean onset time of 544 days, indicating that many adverse events occur during long-term treatment rather than immediately after therapy initiation. The Weibull distribution analysis demonstrated a shape parameter close to 1, corresponding to a random failure-type pattern. This suggests a pattern consistent with a relatively constant hazard over time throughout the treatment period.

Unlike early failure-type adverse reactions that primarily occur shortly after drug initiation, the safety risks associated with givosiran may emerge at any stage of therapy. Consequently, continuous monitoring of biochemical parameters and clinical symptoms should be maintained throughout the entire duration of treatment.

### Sensitivity analysis and robustness of findings

Because several neurological, psychiatric, and renal manifestations identified in this study may also occur as part of the clinical spectrum of acute hepatic porphyria itself, we additionally performed a sensitivity analysis excluding PTs potentially related to the underlying disease manifestations. After exclusion of PTs potentially related to the underlying disease manifestations, the primary disproportionality signals remained statistically significant, supporting the robustness and stability of the main findings. These findings suggest that the observed disproportionality signals were not driven by confounding by disease-related manifestations. The overall consistency between the primary analysis and sensitivity analysis further supports the robustness and stability of the detected positive signals.

### Strengths of the study

This study has several strengths. Primarily, two independent global pharmacovigilance databases were analyzed, which enhances the robustness and generalizability of the detected signals. Furthermore, four disproportionality algorithms were simultaneously applied to improve the reliability of signal detection. In addition, additional analyses—including clinical prioritization and Weibull-based TTO modeling—were performed to further characterize the clinical significance and temporal patterns of adverse events. Together, these methodological approaches provide a more comprehensive assessment of the safety profile of givosiran in real-world clinical practice.

### Clinical implications

The findings of this pharmacovigilance study have several important clinical implications for the management of patients receiving givosiran.

First, although givosiran has demonstrated substantial efficacy in reducing acute attacks of AHP, our analysis highlights several positive signals that warrant careful monitoring in routine clinical practice. These findings support the importance of regular laboratory and clinical monitoring during treatment, particularly in patients with complex comorbidities or prolonged treatment exposure.

Second, the time-to-onset analysis provides additional insights into the temporal patterns of adverse events associated with givosiran. Safety monitoring should be maintained consistently throughout the duration of treatment rather than only during the initiation phase.

Third, the detection of previously underreported or unexpected adverse event signals emphasizes the importance of post-marketing surveillance in evaluating the safety of novel RNA interference therapies. Clinicians should remain aware of these potential risks and report suspected adverse drug reactions to pharmacovigilance systems, thereby contributing to the continuous improvement of drug safety monitoring.

Finally, these findings may help inform individualized risk–benefit assessments when initiating givosiran therapy, particularly in patients with pre-existing hepatic or renal comorbidities. Integrating real-world safety evidence with clinical decision-making may ultimately improve patient management and optimize the long-term safety of givosiran therapy in individuals with acute hepatic porphyria.

### Limitations

Several limitations should be considered when interpreting the results of this study:① Spontaneous reporting systems such as FAERS and VigiAccess are subject to underreporting, reporting bias, and incomplete clinical information, which may affect the accuracy of the findings. ②Disproportionality analysis identifies statistical associations rather than causal relationships. Although sensitivity analyses were performed to reduce potential confounding by indication, some detected adverse events may reflect symptoms of the underlying disease rather than drug-related effects. ③Missing demographic data—particularly age and gender—limited the ability to perform detailed subgroup analyses. Despite these limitations, pharmacovigilance analyses remain an important tool for detecting potential positive signals and generating hypotheses for future research.

## Conclusion

In conclusion, this pharmacovigilance study provides a comprehensive real-world assessment of the safety profile of givosiran using two large international databases, the FAERS and VigiAccess. Multiple consistent positive signals were identified, particularly those related to metabolic, pancreatic, renal, and neurological events, including hyperhomocysteinemia, pancreatitis, renal impairment, and seizure. Several potential adverse events not currently included in the drug labeling were also detected across both databases. These findings highlight the importance of continued pharmacovigilance and long-term clinical monitoring of patients receiving givosiran, especially with respect to homocysteine levels, pancreatic function, renal status, and neurological symptoms. Further prospective studies are warranted to clarify the underlying mechanisms and confirm these safety signals in clinical settings.

## Supplementary Information

Below is the link to the electronic supplementary material.

Supplementary Material 1: Supplementary file 1. Detailed disproportionality analysis results for givosiran-associated adverse events in the FAERS database.

Supplementary Material 2: Supplementary file 2: Detailed disproportionality analysis results for givosiran-associated adverse events in the VigiAccess database.

Supplementary Material 3

## Data Availability

All data used in this study were obtained from publicly accessible pharmacovigilance databases, including the FAERS database (https://fis.fda.gov/extensions/FPD-QDE-FAERS/) and the VigiAccess database (https://www.vigiaccess.org). The data extraction, preprocessing, and analytical procedures are described in detail in the Methods section to facilitate reproducibility.
