# Supplementary material for: Adverse events of givosiran in the treatment of acute hepatic porphyria: a pharmacovigilance study using the FAERS and VigiAccess databases
Source: Orphanet J Rare Dis. 2026 Jun 10;21:284. doi: 10.1186/s13023-026-04446-8 (PMC13487965; doi:10.1186/s13023-026-04446-8)
Supplement: Supplementary file 1 [file 13023_2026_4446_MOESM3_ESM.docx]

Readxl package

ok2 <- data.table(read_xlsx("TTTT.xlsx"))

ok2[,ROR:=((a*d)/(b*c))]

ok2[,RORL:=exp(log(ROR)-1.96*sqrt(1/a+1/b+1/c+1/d))]

ok2[,RORU:=exp(log(ROR)+1.96*sqrt(1/a+1/b+1/c+1/d))]

ok2[,PRR:=(a/(a+b))/(c/(c+d))]

ok2[,XX:=((a*d-b*c)*(a*d-b*c)*(a+b+c+d))/((a+b)*(c+d)*(a+c)*(b+d))]

ok2[,EBGM:=(a*(a+b+c+d))/((a+b)*(a+c))]

ok2[,EBGM05:=exp(log(EBGM)-1.64*(sqrt(1/a+1/b+1/c+1/d)))]

ok2[,IC2:=log2((a*(a+b+c+d))/((a+c)*(a+b)))]

ok2[,GMAE:=((a+b+c+d+2)*(a+b+c+d+2))/((a+b+1)*(a+c+1))]

ok2[,EIC:=log2(((a+1)*(a+b+c+d+2)*(a+b+c+d+2))/((a+b+c+d+GMAE)*(a+b+1)*(a+c+1)))]

ok2[,VIC:=(1/(log(2)))*(1/(log(2)))*((a+b+c+d-3+GMAE)/(3*(1+a+b+c+d+GMAE))+(a+b+c+d-a-b+1)/((a+b+1)*(1+a+b+c+d+2))+(a+b+c+d-a-c+1)/((a+c+1)*(a+b+c+d+3)))]

ok2[,SD:=sqrt(VIC)]

ok2[,BCPNN250:=EIC-2*SD]

ok2[,C025:=IC2-2*SD]

setDT(ok2)

apply(ok2[,.(a,b,c,d)],1,function(x)

{

matrix(x,nrow=2)->inter

chisq.test(inter)$p.value

})->pvalues

ok2[,pvalue:=pvalues]

write.csv(ok2,file = "result .csv")

Forestploter package

Readxl package

list.files()

data <- read_excel("data.xlsx")

data$`ROR(95% CI)` <- paste0(data$ROR,"(",data$lower,"-",data$Upper,")")

data$` ` <- paste(rep(" ", 100), collapse = " ")

data[is.na(data)] <- " "

tm <- forest_theme(base_size = 8,

ci_pch = 15,

ci_col = "#2F4F96",

ci_lty = 1,

ci_lwd = 1.5,

ci_Theight = 0.2,

refline_lwd = 1.5,

refline_lty = "dashed",

refline_col = "red",

#arrow_type = "open",

#arrow_length = 0.05

footnote_cex = 1.1,

footnote_fontface = "italic",

footnote_col = "blue")

p <- forest(data[, c(1, 2, 6, 7)],

est = data$ROR,

lower = data$lower,

upper = data$Upper,

xlim_arrow <- c(0,50),

sizes = 0.6,

ci_column = 4,

ref_line = 1,

xlim = xlim_arrow,

theme = tm)

plot(p)

p_wh <- get_wh(plot = p)

cairo_pdf("forest.pdf",width = p_wh[1], height = p_wh[2])

plot(p)

dev.off()

Dplyr package

Forestploter package

Readxl package

df <- read_xlsx("soc_name_en.xlsx")

colnames(df)

df <- df%>%mutate(SOC=stringr::str_to_title(soc_name_en))

df <- df %>% dplyr::select("SOC",a,ROR,RORL,RORU,pvalue) %>% dplyr::rename(N=a) %>% mutate(pvalue=round(as.numeric(pvalue),2)) %>% mutate(pvalue=case_when(pvalue<0.05~"P<0.05",

TRUE~"P>0.05"))

df$"ROR(95% CI)" <- sprintf("%.2f (%.2f-%.2f)", df$ROR, df$RORL, df$RORU)

df <- df%>%arrange(-N)

df <- df[1:27,]

df$` ` <- paste(rep(" ", 50), collapse = " ")

df[is.na(df)] <- " "

tm <- forest_theme(base_size = 8,

ci_pch = 15, #????????????

ci_col = "#2F4F96", #????????????

ci_lty = 1,

ci_lwd = 1.5, #????????????

ci_Theight = 0.2,

\### ?????????????????????????????????

refline_lwd = 1.5,

refline_lty = "dashed",

refline_col = "red",

\### ??????????????????????????????

footnote_cex = 1.1,

footnote_fontface = "italic",

footnote_col = "blue")

colnames(df)

dt<-df[,c(1,2,7,8,6)]

rightmost<-6

p <- forest(dt, #????????????????????????

est = df$ROR,

lower = df$RORL,

upper = df$RORU,

sizes = 0.6,

ci_column = 4, #???????????????????????????

ref_line = 1, #?????????

#ticks_at=c(1,rightmost),

xlim=c(0,rightmost),

theme = tm)###???????????????

df <- data.table(df)

which(df[,RORU>rightmost])->toadd.arrow

if(length(toadd.arrow))

{

for(indii in toadd.arrow)

{

p<-add_grob(p,row=indii,col=5,part='body',gb_fn='segmentsGrob',

x0=unit(1,'npc')-unit(1,'mm'),

x1=1,

y0=0.5,

y1=0.5,

gp=gpar(fill='#2F4F96',col='transparent'),

arrow=arrow(angle=40,length =unit(0.1, "inches"), ends = "last", type = "closed")

)

}

}

get_wh(p)->wh

jpeg(

filename = "xxx.jpg",

width=wh[1],height=wh[2],

res = 300,

units = "in"

)

plot(p)

dev.off()

Ggplot2 package

Scales package

Ggeasy package

Dplyr package

Readxl package

info<-read_xlsx('data.xlsx')

DDD <- info

names(info)<-c('g','number','ratio')

setDT(info)

info

each<-60

info[,Percentage:=0-ratio*each]

info$g->gs

xpos<-seq(-60,60,by=5)

xlabel<-percent(abs(xpos[xpos<0]/each))

xlabel<-c(xlabel,xpos[xpos>=0])

dat<-melt(info,id.vars='g',measure.vars=c('number','Percentage'))

dat[,g:=ordered(g,gs)]

dat[variable=='number',variable:='n']

dat[variable=='Percentage',variable:='(%)']

a <- levels(dat$g)

DDD$day <- factor(DDD$day,levels =a )

names(DDD)

DDD <- DDD %>%dplyr::rename(value="/%")

p<-ggplot(dat,aes(y=g,x=value,fill=variable))+

geom_bar(stat='identity',position='stack',width=0.6)+

theme_bw()+

labs(x='',y='',fill='',title=' ')+

\# geom_text(aes(x=value+10,label=value),data=dat[value>0],hjust=0)+

theme(legend.position='right',

panel.grid.major.y=element_blank(),

panel.grid.minor.y=element_blank(),

panel.grid.minor.x=element_blank(),

plot.title=element_text(hjust=0.5,size=rel(1.3)))+

scale_x_continuous(breaks=xpos,label=xlabel,expand=expansion(c(0.06,0.06)))+

scale_fill_manual(values=c('#3EB489','#B53E69'))+

theme(panel.grid=element_blank())+

easy_x_axis_title_size(15)+easy_x_axis_labels_size(10)+easy_y_axis_title_size(15)+easy_y_axis_labels_size(15)+

scale_fill_discrete(breaks=c("(%)","n"))

p <- p +

geom_text(data = subset(dat, variable == 'n'),

aes(label = value, y = g, x = value),

hjust = -0.2, vjust = 0.5, size = 3) + # Place "n" labels on the right side

geom_text(data = subset(dat, variable == '(%)'),

aes(label = paste0(round(-1*value*100/each,2), "%"), y = g, x = -abs(value)),

hjust = 1.2, vjust = 0.5, size = 3) # Place "(%)" labels on the left side

ggsave('out.png',p,width=12,height=5.6,dpi=800)

Survminer package

Survival package

Readr package

Readxl package

Survfit package

Dplyr package

list.files()

df <- read_xlsx("data.xlsx")

df <- df%>%mutate(ID = seq(1,by = 1,length.out = nrow(df)))

df$status <- 1

df$Age <- as.factor(df$Age)

df$Time <- as.numeric(df$Time)

fit <- survfit(Surv(Time,status) ~Age,

data = df) #

myplot <- ggsurvplot(fit, data = df,

conf.int = FALSE,

pval = TRUE,

fun = "event",

xlab = "Time(day)",

ylab = "Cumulative",

palette = c("#E7B800", "#2E9FDF", "#FF6666", "#6A0DAD", "#008000"),

ggtheme = theme_bw()

) http://127.0.0.1:39937/graphics/f18c1156-4b07-45af-9ede-12a94b6baa5a.png

ggsave("myplot.pdf", plot = print(myplot), width = 3.3, height = 2.2, dpi = 1000)

Fitdistrplus package

weibull_fit <- fitdist(time, "weibull")

weibull_loglik <- weibull_fit$loglik

weibull_AICc <- AICc(weibull_loglik, length(time), 2)

weibull_n2logL <- -2 * weibull_loglik

lnorm_fit <- fitdist(time, "lnorm")

lnorm_loglik <- lnorm_fit$loglik

lnorm_AICc <- AICc(lnorm_loglik, length(time), 2)

lnorm_n2logL <- -2 * lnorm_loglik

gamma_fit <- fitdist(time, "gamma")

gamma_loglik <- gamma_fit$loglik

gamma_AICc <- AICc(gamma_loglik, length(time), 2)

gamma_n2logL <- -2 * gamma_loglik

exp_fit <- fitdist(time, "exp")

exp_loglik <- exp_fit$loglik

exp_AICc <- AICc(exp_loglik, length(time), 1)

exp_n2logL <- -2 * exp_loglik

results <- data.frame(

Distribution = c("Weibull", "Log-Normal", "Gamma", "Exponential"),

LogLikelihood = c(weibull_loglik, lnorm_loglik, gamma_loglik, exp_loglik),

AICc = c(weibull_AICc, lnorm_AICc, gamma_AICc, exp_AICc),

n2logL = c(weibull_n2logL, lnorm_n2logL, gamma_n2logL, exp_n2logL)

)

print(results)
